# Limitations in Chest X-Ray Interpretation by Vision-Capable Large Language Models, Gemini 1.0, Gemini 1.5 Pro, GPT-4 Turbo, and GPT-4o

**DOI:** 10.3390/diagnostics16030376

**Published:** 2026-01-23

**Authors:** Chih-Hsiung Chen, Chang-Wei Chen, Kuang-Yu Hsieh, Kuo-En Huang, Hsien-Yung Lai

**Affiliations:** 1 Department of Critical Care Medicine, Mennonite Christian Hospital, Hualien 970472, Taiwan; foolman.chen@gmail.com (C.-H.C.); hsiehkuangyu@yahoo.com.tw (K.-Y.H.); doc10455@gmail.com (K.-E.H.); 2 Department of Emergency, Mennonite Christian Hospital, Hualien 970472, Taiwan; silver.hawk@msa.hinet.net; 3 Department of Anesthesiology, Da-Chien Health Medical System, Miaoli 36052, Taiwan

**Keywords:** chest X-rays, GPT, Gemini, vision-capable, performance, language model

## Abstract

**Background/Objectives**: Interpretation of chest X-rays (CXRs) requires accurate identification of lesion presence, diagnosis, location, size, and number to be considered complete. However, the effectiveness of large language models with vision capabilities (LLMs) in performing these tasks remains uncertain. This study aimed to evaluate the image-only interpretation performance of LLMs in the absence of clinical information. **Methods**: A total of 247 CXRs covering 13 diagnostic categories, including pulmonary edema, cardiomegaly, lobar pneumonia, and other conditions, were evaluated using Gemini 1.0, Gemini 1.5 Pro, GPT-4 Turbo, and GPT-4o. The text outputs generated by the LLMs were evaluated at two levels: (1) primary diagnosis accuracy across the 13 predefined diagnostic categories, and (2) identification of key imaging features described in the generated text. Primary diagnosis accuracy was assessed based on whether the model correctly identified the target diagnostic category and was classified as fully correct, partially correct, or incorrect according to predefined clinical criteria. Non-diagnostic imaging features, such as posteroanterior and anteroposterior (PA/AP) views, side markers, foreign bodies, and devices, were recorded and analyzed separately rather than being incorporated into the primary diagnostic scoring. **Results**: When fully and partially correct responses were treated as successful detections, vLLMs showed higher sensitivity for large, bilateral, multiple lesions and prominent devices, including acute pulmonary edema, lobar pneumonia, multiple malignancies, massive pleural effusions, and pacemakers, all of which demonstrated statistically significant differences across categories in chi-square analyses. Feature descriptions varied among models, especially in PA/AP views and side markers, though central lines were partially recognized. Across the entire dataset, Gemini 1.5 Pro achieved the highest overall detection rate, followed by Gemini 1.0, GPT-4o, and GPT-4 Turbo. **Conclusions**: Although LLMs were able to identify certain diagnoses and key imaging features, their limitations in detecting small lesions, recognizing laterality, reasoning through differential diagnoses, and using domain-specific expressions indicate that CXR interpretation without textual cues still requires further improvement.

## 1. Introduction

Artificial intelligence (AI), particularly in areas such as deep learning and neural networks, has seen gradual adoption in the healthcare field over the past few years. Notable applications include diagnostic imaging in medicine, the development of models predicting mortality rates for specific diseases and providing health-related information [1,2,3,4]. Currently, multimodal large language models (LLMs) are demonstrating increasing utility across diverse domains beyond medicine. In education, they have been shown to support personalized vocabulary learning by dynamically integrating text and images, drawing on cognitive principles such as dual coding to enhance memory retention and learner engagement [5,6]. The success of large language models, such as the generative pretrained transformer (GPT) that emerged in 2022 [7] and Google Gemini, which was deployed in December 2023 [8], has begun to open new fields of application in healthcare.

Various studies have tested the utility of these LLMs in medical writing exams, demonstrating significant improvements in natural language processing. These models have performed at or near the passing threshold for various medical exams without specialized training [9,10]. With the introduction of vision capabilities, there has yet to be an evaluation of these vision-capable LLMs in interpreting medical images.

Unlike written examinations that yield binary outcomes, a complete medical image interpretation is considered achieved only when the presence of a lesion, its diagnosis, location, size, and number are all accurately identified. Vision-capable AI may detect lesions but still generate errors due to unfamiliarity with clinical reasoning and medical terminology. In the field of CXRs, the ability to interpret images without prior knowledge of a patient’s medical history represents a critical skill for evaluating a radiologist’s true diagnostic competence.

This study compares chest X-ray interpretations across multiple diagnostic categories using general-purpose vision-language models, with the aim of characterizing their image-only performance and identifying systematic limitations. Rather than serving as a diagnostic benchmark or a measure of clinical readiness, we adopt an image-only failure mode analysis framework to examine how these models succeed, partially succeed, or fail under controlled and favorable conditions. By focusing on recurrent error patterns and boundary cases, this work seeks to inform future evaluation designs and guide cautious downstream use, rather than to establish diagnostic reliability.

## 2. Materials and Methods

**Figure 1:**
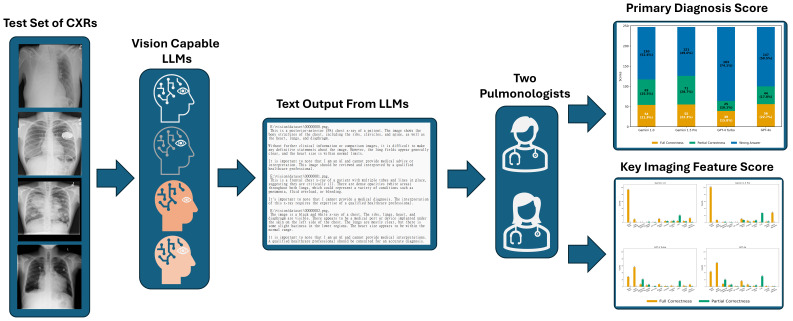
Flowchart of the Study. Abbreviations: CXR: chest X-rays, LLM: large language model.

The entire research workflow is outlined in Figure 1, with the following sections providing detailed explanations of each step in the process. This study complies with the requirements of the Checklist for Artificial Intelligence in Medical Imaging (CLAIM): 2024 Update, and the relevant information is provided in the Supplemental Material Section S1 for reference.

### 2.1. Image Dataset and LLMs Selection

We selected the National Institutes of Health Chest X-ray Dataset (NIHCXR) for this study. The dataset contains 112,120 frontal chest radiographs from 30,805 unique patients and is publicly available under the Creative Commons CC0: Public Domain license [11]. NIH-provided labels were used only for initial case retrieval and were not treated as the reference standard for this study.

#### 2.1.1. Chest X-Rays Selection and Randomization

Chest X-rays with obvious and typical radiographic findings were selected through direct image-based review by two board-certified pulmonologists with expertise in chest radiograph interpretation. Final category assignment (ground truth) was determined by clinician consensus based on visual assessment of each image, rather than reliance on NIH-provided labels.

To construct a clean and unambiguous test set, images were selected to represent a single, dominant primary target within 1 of 13 predefined diagnostic categories. Images showing overlapping conditions across categories were excluded (e.g., cardiomegaly with concurrent acute pulmonary edema, or unilateral massive pleural effusion with contralateral lobar pneumonia). Images containing additional confounding diagnoses, such as pneumothorax or pneumoperitoneum, were also excluded.

Only cases demonstrating clear and characteristic imaging features were included. Any image for which there was even minor uncertainty or disagreement between the two reviewers was excluded and replaced. Because the dataset was intentionally restricted to obvious and typical cases, full clinician consensus was readily achieved, and no adjudication process was required.

These were 20 images of acute pulmonary edema, 20 of cardiomegaly, 10 of hiatal hernia, 15 of lobar pneumonia, 15 of pacemaker, 5 of port-a-cath, 10 of peripherally inserted central catheter (PICC), pleural effusion with 15 of minimal, 10 of small, 15 of moderate, and 10 of massive amount, malignancy with 15 of central distributed lesion, 22 of single lesion, and 30 of multiple lesion, 5 of diaphragm elevation, and 30 with no findings, totaling 247 images across 13 categories. To prevent potential identification of filenames and their associated diagnoses through online searches, the selected CXRs were renamed randomly, ranging from 00000000.png to 00000246.png in the final test set. See “Test dataset” in the Supplemental Material Section S2.

#### 2.1.2. Selection of Vision-Capable LLMs

We employed four vision-capable LLMs to interpret the CXRs, using the following models and their respective application programming interface (API) versions: Gemini Vision Pro (version 1.0, with model name “gemini-pro-vision”), Gemini 1.5 Pro (model name “gemini-1.5-pro-latest”), GPT-4 Turbo (model name “gpt-4-turbo”), and GPT-4o (model name “gpt-4o”). Their version dates were as follows: Gemini 1.5 Pro and GPT-4o dated 10 June 2024, Gemini Vision Pro dated 12 June 2024, and GPT-4 Turbo dated 11 June 2024.

#### 2.1.3. Test Setting

The prompt used uniformly across all models was “Please describe the image.” Text outputs generated by the LLMs were saved as text files. Model inference was performed using the official ChatGPT and Gemini APIs with the default inference parameters provided by each platform at the time of evaluation. Specifically, no manual adjustments were made to temperature, top_p, or max_tokens, as our objective was to assess model performance under standard, out-of-the-box usage conditions rather than under optimized or customized settings. Images were provided in base64-encoded format for the ChatGPT API and as original PNG files for the Gemini API. Each image was processed once per model, without repeated sampling.

### 2.2. How to Describe the Lesions in CXR

#### 2.2.1. Criteria for a Complete Lesion Description

In clinical practice, when interpreting a lesion on a CXR, several aspects must be evaluated to achieve a complete lesion description. The first step is to determine whether a lesion is visible. The interpreter must then accurately describe its location on the right or left side, the number of lesions, and their size. Finally, the diagnosis of the lesion, or an appropriate differential diagnosis, should be provided. Only when all these aspects are correctly identified can the interpretation be considered a complete assessment of the CXR lesion.

If any of these elements are incorrect, the interpretation should not be regarded as fully correct. For example, if the ground truth indicates right lobar pneumonia but the model outputs pneumonia in the left lobe, it cannot be considered correct. However, in vision-capable AI, such an error may suggest that the model detected the lesion but misclassified its laterality due to training limitations. Therefore, classifying each response as fully correct, partially correct, or incorrect provides a more appropriate framework for evaluating the performance of LLMs.

#### 2.2.2. Definitions of Fully Correct, Partially Correct, and Incorrect Answers

The definition of a fully correct answer included three scenarios: a definite diagnosis with the correct site, inclusion of the correct diagnosis in the differential diagnosis with the correct site, and descriptions indicating normal findings without mentioning any abnormalities. Partial correctness included mentioning the lesion with the correct diagnosis but the wrong site, or the correct site but the wrong diagnosis. Other cases included detection of the lesion without providing definite or differential diagnosis, mentioning the lesion with the wrong site or wrong diagnosis, or the correct diagnosis and site but incorrect magnitude. Incorrect answers indicated that the LLMs failed to detect the lesions, as they did not mention the corresponding findings in their responses, with the primary diagnosis used as the reference standard.

For the evaluation criteria, any response that mentioned the presence of a lesion was considered partially correct, even if multiple descriptive errors were present. For example, a case described as “a port-a-cath at left upper lung” but output by the model as “a pacemaker at right lung” was classified as partially correct because the model identified the presence of a device, despite containing two descriptive errors. 

#### 2.2.3. Primary Diagnosis and Key Imaging Features Scoring

The evaluation focused on two main aspects: the accuracy of the primary diagnosis and the extraction of pertinent imaging features from the generated description text.

For the primary diagnosis, since a reference standard was available, the scoring assessed whether each LLM provided the correct main diagnosis for a given CXR. For cases with a confirmed primary diagnosis, the evaluation followed the criteria for a complete lesion description, which included the presence of a lesion, its laterality, size, and the accuracy of the definite or differential diagnosis. Each case was therefore classified as fully correct, partially correct, or incorrect.

In contrast, the evaluation of imaging feature scores did not rely on a fixed reference standard. Instead, the assessment was based on the descriptive text generated by each model, which included mentions of other lesions, devices, letters, or imaging findings visible on the CXR. Because the imaging features identified by different models were not entirely consistent, the most frequently reported elements were defined as the key imaging features for statistical analysis in this study. These included the posterior–anterior or anterior–posterior (PA/AP) view, left/right side markers, other letters or numbers appearing in the image, foreign bodies, identification of the patient as adult or child and as male or female, surgical clips or implants, monitoring wires or leads, and intravenous lines, ports, or devices. Each description was rated as correct or partially correct, and the total number of correctly identified features was summed for subsequent analysis.

### 2.3. Scoring the LLMs

The text outputs generated by the LLMs were subsequently analyzed by two pulmonologists. The first pulmonologist independently reviewed the LLM-generated texts and, based on professional expertise in chest X-ray interpretation, compared each output with the corresponding image. The second pulmonologist independently reviewed the same outputs and served as a verifier. Full consensus was achieved after verification; therefore, formal inter-rater agreement statistics were not calculated. To minimize potential bias, the scoring pulmonologists were blinded to model identity. All model outputs were exported as plain text files and labeled using anonymized numeric identifiers, with no information indicating the source large language model.

### 2.4. Statistical Analysis and Software

#### 2.4.1. Statistical Analysis Methods

The analysis was divided into two performance levels: fully correct and fully correct plus partially correct. The latter was defined as cases in which the model successfully detected the lesion but failed to meet all diagnostic criteria. This outcome may occur because LLMs can identify visual abnormalities but still generate inaccurate descriptions due to limited familiarity with clinical reasoning and medical terminology. The chi-square test was applied to infer the statistical significance of performance differences. Analyses were conducted into the overall performance combining all four LLMs. To facilitate interpretation, the 13 primary diagnostic categories were grouped according to their radiographic characteristics as follows:

Large-sized lesions included acute pulmonary edema, lobar pneumonia, and cardiomegaly. Acute pulmonary edema affects both lung fields, whereas lobar pneumonia involves a localized lesion within a single lung field. Cardiomegaly is located within the mediastinum and partially obscures the medial portions of both lower lung fields. These categories were used to evaluate the model’s ability to detect lesions of varying sizes. Lesion number consisted of malignancies with a single lesion versus multiple lesions, allowing assessment of the model’s sensitivity to the number of lesions present in an image. Mediastinal lesions included malignancy with a central distribution and hiatal hernia. These conditions were used to evaluate the model’s ability to detect findings within or adjacent to the mediastinum. Devices included pacemaker, port-a-cath, and PICC. These devices have distinct radiographic appearances and strong image contrast. The pacemaker is generally larger than the port-a-cath, and both exhibit line-like structures on chest radiographs, whereas the PICC primarily represents a linear structure alone. Pleural effusion and diaphragm elevation were analyzed to assess the model’s detection capability for pleural effusion. Diaphragm elevation exhibits radiographic features like those between small and moderate pleural effusion, although they are pathophysiologically distinct conditions.

Finally, a model performance comparison was conducted using the primary diagnosis scores of the four LLMs. A chi-square test was applied to evaluate and compare the diagnostic performance among these models.

#### 2.4.2. Software

The analyses were performed using Python 3.8 with the statistical functions provided in the SciPy 1.10.1 package. All graphical outputs were generated using Matplotlib version 3.7.5.

## 3. Results

### 3.1. Results for Primary Diagnosis

#### 3.1.1. Scores of Four LLMs in Primary Diagnosis

Detailed per-category results, including the total number of cases and the proportions of fully and partially correct interpretations, are provided in Table 1, while overall model-level performance with corresponding 95% confidence intervals is summarized in Supplementary Material Section S3.

**Figure 2:**
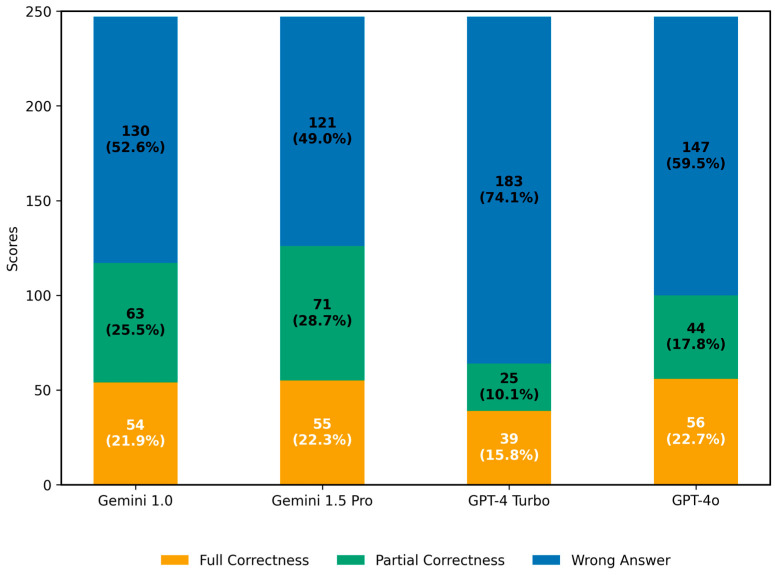
Total Scores in Primary Diagnosis. The x-axis shows the four models: Gemini 1.0, Gemini 1.5 Pro, GPT-4 Turbo, and GPT-4o. The y-axis represents the total score, with a cumulative total of 247 for each model. The numbers displayed at the center of each colored bar indicate the corresponding score, while the values in parentheses represent the percentage.

In primary diagnosis, the four LLMs fully correctly identified 54, 55, 39, and 56 cases in the order of Gemini 1.0, Gemini 1.5 Pro, GPT-4 Turbo, and GPT-4o, respectively. Partial correctness was observed in 63, 71, 25, and 44 cases. See Figure 2.

#### 3.1.2. Analysis of Partial Correctness in Primary Diagnosis

**Figure 3:**
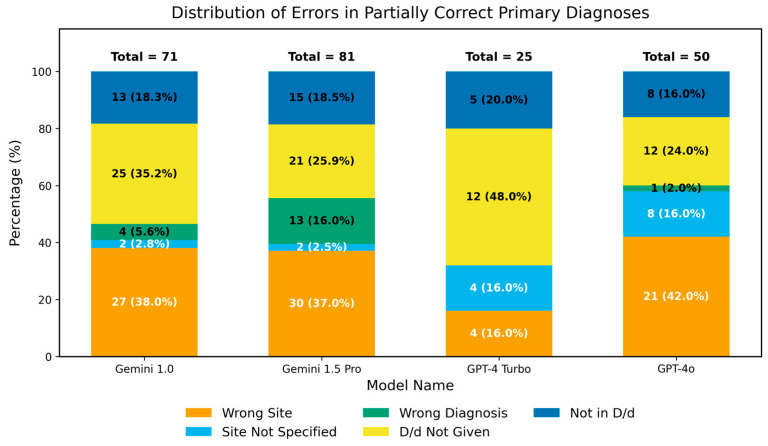
Distribution of Errors in Partially Correct Primary Diagnoses. The number above each bar in the chart represents its value. Wrong Site: confusion between the right and left sides; Site Not Specified: the lesion is mentioned without specifying the exact location; Wrong Diagnosis: the lesion is mentioned but the diagnosis is incorrect; D/d (Differential Diagnosis) Not Given: the lesion is mentioned without providing a definitive or differential diagnosis; Not In D/d: the differential diagnoses provided does not match the primary diagnosis.

In the primary diagnosis analysis, some partially correct responses contained left-right location errors: Gemini 1.0 Pro had 27 such cases, Gemini 1.5 Pro had 30, GPT-4 Turbo had 4, and GPT-4o had 21. There were 2, 2, 4, and 8 cases, respectively, in which a lesion was mentioned but its specific location was not indicated. The numbers of incorrect diagnoses were 4, 13, 0, and 1. There were 25, 21, 12, and 12 cases in which a lesion was mentioned without providing a definitive or differential diagnosis. Additionally, there were 13, 15, 5, and 8 cases in which both a lesion and a differential diagnosis were mentioned, but the differential diagnosis did not correctly identify the primary diagnosis (Figure 3).

For the evaluation criteria, any response that mentioned the presence of a lesion was considered partially correct, even if multiple descriptive errors were present. Therefore, the cumulative count of such cases may exceed the total partial correctness scores as shown in Figure 2.

#### 3.1.3. Scores Across Thirteen Categories

Scores of full correctness were as follows: acute pulmonary edema (7, 6, 0, 6, in the order of Gemini 1.0, Gemini 1.5 Pro, GPT-4 Turbo, and GPT-4o, respectively), cardiomegaly (1, 3, 1, 0), lobar pneumonia (1, 3, 0, 1), pacemakers (10, 8, 13, 6), port-a-cath (0, 0, 0, 2), pleural effusion (3, 6, 0, 4), malignancy with single lesion (0, 0, 0, 1), malignancy with multiple lesions (4, 0, 1, 5), diaphragm elevation (0, 0, 0, 1), and normal chest images (28, 29, 24, 30). No correct identifications were made for hiatal hernia, PICC, or malignancy with central distribution.

**Figure 4:**
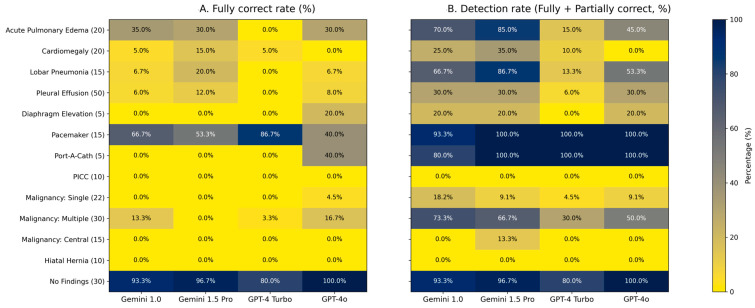
Model performance across thirteen diagnostic categories. (**A**) shows the fully correct rate (%) for each diagnostic category, and (**B**) shows the detection rate (%), defined as the proportion of cases classified as either fully or partially correct. Numbers in parentheses adjacent to each category on the y-axis denote the total number of images in that category. Rows correspond to diagnostic categories, with the total number of images in each category indicated in parentheses, and columns correspond to the evaluated models (Gemini 1.0, Gemini 1.5 Pro, GPT-4 Turbo, and GPT-4o). Color intensity represents the percentage value on a continuous scale from 0% to 100%, as indicated by the color bar. Exact percentage values are annotated within each cell to facilitate direct comparison across models and categories.

Partial accuracy was as follows: acute pulmonary edema (7, 11, 3, 3), cardiomegaly (4, 4, 1, 0), lobar pneumonia (9, 10, 2, 7), pacemakers (4, 7, 2, 9), port-a-cath (4, 5, 5, 3), pleural effusion (12, 9, 3, 11), malignancy with central distribution (0, 2, 0, 0), malignancy with single lesion (4, 2, 1, 1), malignancy with multiple lesions (18, 20, 8, 10), and diaphragm elevation (1, 1, 0, 0). No partial accuracy was observed for hiatal hernia, PICC, or normal chest images. The model performance across thirteen diagnostic categories is depicted in Figure 4.

#### 3.1.4. Analysis for Pleural Effusion

**Figure 5:**
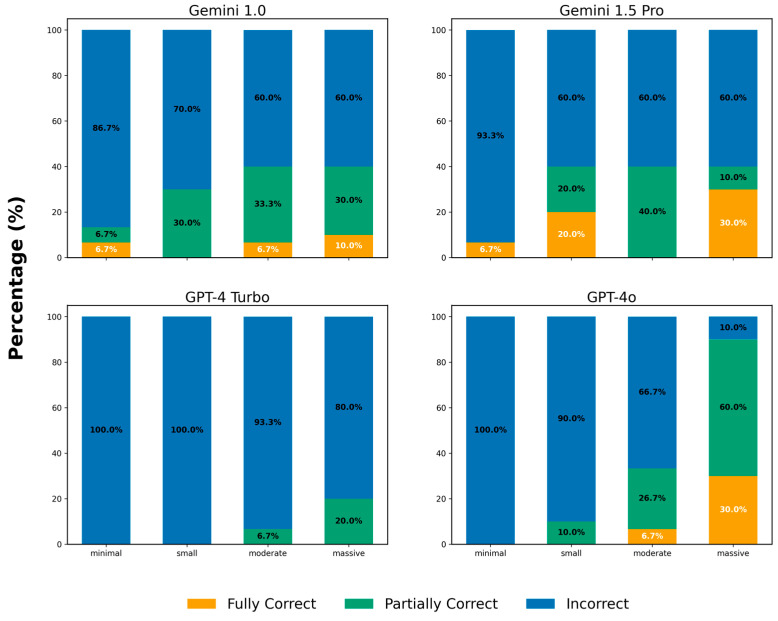
Scores in Different Amount of Pleural effusion. The y-axis represents the percentage of correct responses, while the x-axis represents the volume of pleural effusion: minimal, small, moderate, and massive. The numbers displayed at the center of each colored bar indicate the corresponding percentage.

In the pleural effusion category, Gemini 1.0 correctly described 1 case of minimal effusion, 0 cases of small effusion, 1 case of moderate effusion, and 1 case of massive effusion. For Gemini 1.5 Pro, the corresponding numbers of correctly described cases were 1, 2, 0, and 3, respectively. GPT-4 Turbo was incorrect in all cases, whereas GPT-4o correctly described 0, 0, 1, and 3 cases in the same order. Partial correctness was observed in 1, 3, 5, and 3 cases for minimal, small, moderate, and massive effusions, respectively, in Gemini 1.0. For Gemini 1.5 Pro, the corresponding numbers were 0, 2, 6, and 1. GPT-4 Turbo had 0, 0, 1, and 2 partially correct cases, while GPT-4o had 0, 1, 4, and 6, respectively. Please refer to Figure 5.

### 3.2. Results for Key Imaging Features

#### 3.2.1. Scores for Key Imaging Features

**Figure 6:**
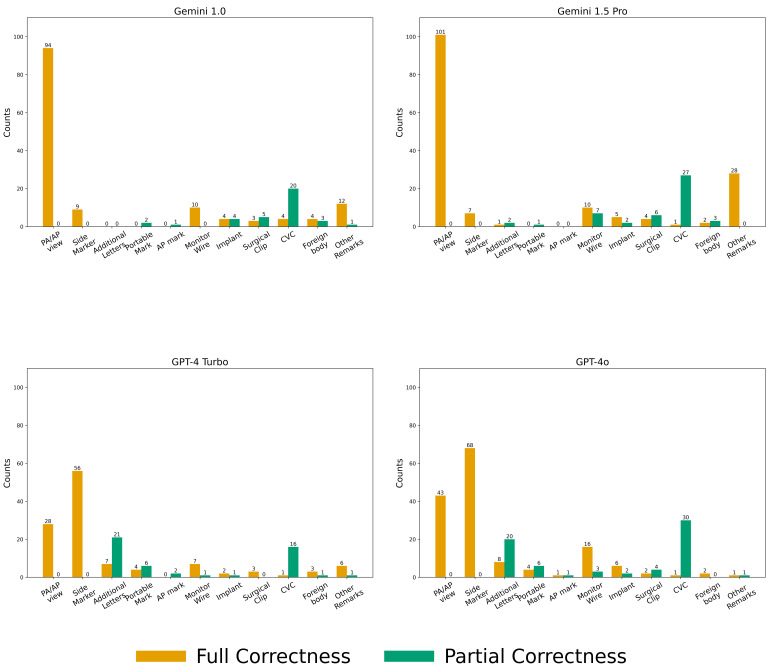
Details in Key Imaging Feature Score Among Models. The number above each bar in the chart represents its value. Abbreviation: PA/AP: posterior–anterior/anterior–posterior; Side marker: “R” or “L”, or any other letters indicating the patient’s side on a CXR; Additional letters: any letters appearing on the image, which may be in normal orientation, reversed left-right, flipped upside down, or both flipped and reversed; Portable mark: the word “PORTABLE” in the image; AP mark: the letters “AP” in the image; CVC: central venous catheter; Foreign body: anything outside the body, such as a necklace, clothes zipper, or earrings; Other remarks: indicating breast shadows or distinguishing between an adult or child.

In the key imaging feature description, Gemini 1.0 fully correctly described 140 features, Gemini 1.5 Pro described 159, GPT-4 Turbo described 117, and GPT-4o described 152. Partial correctness was observed in 36, 48, 49, and 67 cases, respectively. Specific feature identification varied across several attributes, including the posterior–anterior/anterior–posterior (PA/AP) view (94, 101, 28, and 43), side markers (9, 7, 56, and 68), additional letters (0, 1, 11, and 13), external monitoring wires and leads (10, 10, 7, and 16), surgical implants (4, 5, 2, and 6), surgical clips (3, 4, 3, and 2), central lines, ports, or devices (4, 1, 1, and 1), and identification of female and adult subjects (12, 28, 6, and 1). See Figure 6.

#### 3.2.2. Analysis of Partial Correctness in Central Venous Catheter Diagnosis

**Figure 7:**
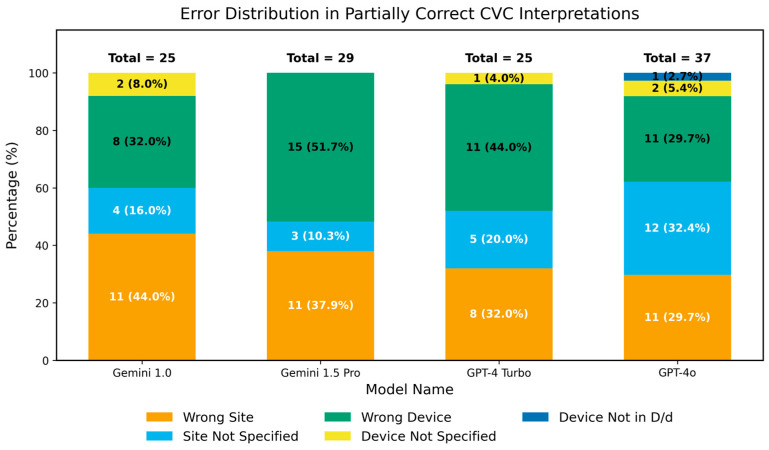
Error Distribution in Partially Correct CVC Interpretations. The number above each bar in the chart represents its value. Wrong Site refers to confusion between the right and left sides; Site Not Specified indicates that the lesion is mentioned without specifying the exact location; Wrong Device means the device is mentioned, but the diagnosis is incorrect; Device Not Specified refers to the device being mentioned without providing a name; and Device Not In D/d (Differential Diagnosis) means the differential diagnosis provided does not match the device name.

In the analysis of key imaging features, we found that all models achieved high detection scores for central venous catheters (CVCs), although these were often only partially correct. Therefore, we conducted a focused analysis of these findings. Left–right location errors were observed in 11 cases each for Gemini 1.0 and Gemini 1.5 Pro, 8 for GPT-4 Turbo, and 11 for GPT-4o. Specific device locations were not indicated in 4, 3, 5, and 12 cases, respectively. Incorrect device names were given in 8, 15, 11, and 11 cases, while 2, 0, 1, and 2 instances involved descriptions mentioning only the device without its name. Additionally, a differential diagnosis failed to identify the correct device name in 0, 0, 0, and 1 case, respectively. See Figure 7. Consistent with the approach used in Figure 2, the cumulative count may be greater than the total number of partially correct cases because a single partially correct lesion can involve more than one descriptive error.

### 3.3. Statistical Analysis

#### 3.3.1. Statistical Analysis for Five Major Groups

A total of five major groups were analyzed to evaluate the diagnostic performance of all LLMs. Each group represented clinically distinct imaging characteristics designed to assess lesion size, lesion number, mediastinal involvement, device recognition, and pleural findings.

In the large-sized lesion group, which included acute pulmonary edema, lobar pneumonia, and cardiomegaly, a statistically significant difference was observed among the three conditions for fully correct responses (χ^2^ = 12.40, *p* = 0.0020). The fully correct rates were acute pulmonary edema (23.8%), lobar pneumonia (8.3%), and cardiomegaly (6.2%). When both fully and partially correct responses were treated as successful detections, the difference remained significant (χ^2^ = 28.52, *p* < 0.0001). The detection rates followed a similar pattern, with lobar pneumonia (55.0%), acute pulmonary edema (53.8%), and cardiomegaly (17.5%).

In the lesion number group, which compared malignancy with a single lesion and malignancy with multiple lesions, a significant difference was found in fully correct responses (χ^2^ = 3.91, *p* = 0.0480), with the multiple-lesion group (8.3%) outperforming the single-lesion group (1.1%). When both fully and partially correct responses were considered, the difference became highly significant (χ^2^ = 42.22, *p* < 0.0001). The detection rates were multiple (55.0%) and single (10.2%), indicating greater sensitivity of LLMs for multiple lesions compared with solitary findings.

In the mediastinal lesion group, including malignancy with central distribution and hiatal hernia, all four LLMs demonstrated complete failure of recognition. None of the models produced any fully correct responses, and all yielded zero detections for hiatal hernia. Only two partially correct responses were generated by Gemini 1.5 Pro in the malignancy-with-central-distribution category. Because all observed counts were zero or near zero, no valid chi-square test could be performed.

In the device group, comprising pacemaker, port-a-cath, and PICC, statistically significant differences were observed among the three device types when only fully correct responses were analyzed (χ^2^ = 47.14, *p* < 0.0001). The fully correct rates were pacemaker (61.7%), port-a-cath (10.0%), and PICC (0.0%). When fully and partially correct responses were combined, the difference remained highly significant (χ^2^ = 111.50, *p* < 0.0001). The detection rates were pacemaker (98.3%), port-a-cath (95.0%), and PICC (0.0%), indicating that LLMs exhibited superior recognition performance for large, high-contrast implanted devices.

In the pleural effusion group, which included minimal, small, moderate, and massive pleural effusion as well as diaphragm elevation, significant differences were found across the five subcategories. For fully correct responses, the chi-square test showed significance (χ^2^ = 10.36, *p* = 0.0348). The fully correct rates were massive (17.5%), small (5.0%), diaphragm elevation (5.0%), minimal (3.3%), and moderate (3.3%). When fully and partially correct detections were combined, the difference became more pronounced (χ^2^ = 26.97, *p* < 0.0001). The detection rates were massive (47.5%), moderate (30.0%), small (20.0%), diaphragm elevation (15.0%), and minimal (5.0%), demonstrating that larger effusion volumes were more reliably identified by the models, whereas smaller effusions and diaphragm elevation were often underrecognized. See Supplementary Material Section S4.

#### 3.3.2. Model Performance Comparison

A chi-square test of independence demonstrated no statistically significant difference among the four LLMs in the distribution of fully correct responses (χ^2^ = 4.79, *p* = 0.188). As summarized in Supplementary Material Section S3, Gemini 1.5 Pro, GPT-4o, and Gemini 1.0 showed comparable proportions of fully correct interpretations, whereas GPT-4 Turbo exhibited a lower rate.

When fully and partially correct responses were combined and treated as successful detections, a statistically significant difference emerged among the models (χ^2^ = 37.58, *p* < 0.0001). Under this criterion, Gemini 1.5 Pro achieved the highest detection rate, followed by Gemini 1.0, GPT-4o, and GPT-4 Turbo. For detailed Table, see Supplementary Material Section S3.

## 4. Discussion

Currently, there are several customized AI systems available to assist with medical image interpretation, such as CE-marked AI-based software [12,13], and for certain clinical conditions, like heart failure, endotracheal tube positioning, and COVID-19 [14,15,16], which achieve specific results. To contextualize our findings, prior studies of task-optimized, CE-marked CXR AI systems have reported higher performance on specific reporting dimensions than GPT-4o. For example, a recent observer study showed that a CE-marked CXR system achieved acceptable interpretation rates of approximately 60–62%, compared with approximately 43–45% for GPT-4o, and substantially fewer location errors (~76–77% vs. ~36%). Such results underscore that specialized, regulated systems may offer more stable performance for narrowly defined tasks. However, direct comparison with the present image-only failure mode analysis is limited by differences in datasets, case selection, and evaluation criteria [13]. In our study, we used four publicly available LLMs with visual capabilities, Gemini 1.0, Gemini 1.5 pro, GPT-4 turbo and GPT-4o.

In comparison of primary scores among LLMs, we observed that the models exhibited relatively better detection capabilities for bilateral lesions such as acute pulmonary edema, malignancy with bilateral distribution, and lobar pneumonia. Pacemaker and port-a-cath detection rates were the highest, likely due to the distinctiveness of these devices. Conversely, the detection rate for PICC lines was not as high, possibly due to their relatively thin and small scale. The models also struggled significantly with detecting lesions in mediastinum. They exhibited almost no detection capability for hiatal hernia and malignancy with central distribution.

We classified pleural effusion into four categories: minimal pleural effusion, which indicates costophrenic angle blunting; small amount, which indicates the disappearance of the unilateral diaphragm; moderate pleural effusion, which indicates a lesion covering more than half of the lung field; and massive pleural effusion, which indicates that the entire lung field appears white [17]. Both minimal and small amounts show poor detection rates. A similar analogy applies to diaphragm elevation [18], indicating that future advancements in medical vision capability will require further algorithmic improvements to detect these conditions.

For the “no finding” category, most scores were achieved by describing anatomical structures as normal. However, cases where the models directly interpreted the image as normal were relatively few. In this aspect, the Gemini models demonstrated significant confidence in diagnosing normal CXRs. It is important to note that while publicly available LLMs provide similar interpretations, they also have the potential to spread misinformation and exacerbate the misuse of AI in medical imaging due to a lack of accountability [19]. This highlights the need for medical professionals to be involved in making accurate diagnoses [20].

In imaging feature interpretation, the Gemini models showed a notable advantage in imaging feature description tasks, particularly in distinguishing between PA/AP views, detecting CVCs, and identifying female breast shadows. On the other hand, GPT models emphasized the detection of side markers and other letters to annotate the image and demonstrated competency in detecting CVCs. Both models demonstrated higher detection rates for pacemakers.

We introduced the concept of partial correctness because we observed that LLMs were sometimes able to detect the presence of lesions, even when the final interpretation was incomplete or inaccurate. In such cases, the models might assign an incorrect diagnosis, describe the lesion on the wrong side, or include the finding within a list of differential diagnoses that did not contain the correct answer. Although some authors have interpreted these outputs as manifestations of artificial hallucinations [21,22], we suggest that, with further training or more comprehensive differential diagnosis generation, there may be room for improvement [23]. Nevertheless, it is important to emphasize that while partial correctness was treated as a successful detection in this study for analytical purposes, such outputs could pose substantial risks in real-world medical contexts. Patients seeking medical advice based on partially correct yet confident interpretations may experience diagnostic confusion, delayed treatment, or even medico-legal disputes. This concern is particularly relevant in medical education settings, where trainees or junior clinicians may overtrust fluent multimodal outputs in the absence of sufficient domain expertise.

A recurring issue observed across these models is left–right confusion. In clinical practice, radiographic terminology is based on the patient’s anatomical left and right sides, such that the right side of the image corresponds to the patient’s left side, and vice versa. Whether this reflects a fundamental limitation of current multimodal alignment strategies or a bias introduced during data annotation and training remains an open question. Despite this well-established convention, vision-capable models frequently misidentify laterality, and addressing this limitation could improve diagnostic accuracy and overall performance [24]. Similar reductions in performance have been noted in prior studies involving natural language generation for chest X-ray interpretation, including CXAS U-Net and BioGPT with a BLIP-2 modular architecture [25].

In interpreting a CXR, providing a list of potential differential diagnoses can significantly enhance clinical assessment [26], particularly in the absence of corroborating clinical data. For this study, we adopted a scoring approach where the LLM was awarded points if its proposed differential diagnosis included the correct diagnosis. Our analysis reveals that offering a more comprehensive list of differential diagnoses could substantially improve the clinical utility of these models, effectively supporting clinicians’ diagnostic reasoning. This approach allows for a broader consideration of potential conditions, which may be particularly valuable in complex or ambiguous cases, and may improve the usability of AI-assisted large language models for radiologists [27]. Related studies have shown that similar mechanisms can allow a frozen radiology vLLM to self-correct and produce more accurate [28] and consistent chest X-ray reports, illustrating a potential framework for clinician–AI collaboration in radiology report composition and suggesting avenues for future clinical application [29].

### Limitations

The field of chest X-ray interpretation is broad and heterogeneous. In this study, we intentionally restricted image selection to cases with obvious and typical radiographic findings, excluding subtle, borderline, or ambiguous presentations. This design choice was intended to reduce confounding factors and enable a controlled comparison of image-only performance across models. However, such case enrichment may inflate apparent diagnostic performance and limit the generalizability of the results to real-world clinical scenarios, where findings are often subtle, overlapping, or equivocal. In addition, abnormalities such as rib fractures, subdiaphragmatic conditions, and severe thoracic emergencies (e.g., pneumothorax and pneumomediastinum) were not included in the analysis. Furthermore, no clinical history was provided for each CXR. While this approach minimized potential bias from clinical context, it also limited the assessment of diagnostic accuracy in scenarios where clinical information is available and routinely used in practice [30]. Although previous studies have suggested that extracting specific regions from whole CXRs can improve diagnostic accuracy [31], the present study used entire CXRs as inputs without any image preprocessing. The evaluated models represent specific versions available at the time of testing, and given the rapid pace of development in LLMs, the observed performance may not generalize to subsequent model releases. The uneven sample sizes across categories, particularly in smaller groups, may limit statistical power and the stability of category-specific estimates. Lastly, as this study was based on a single dataset (NIHCXR), the findings may not be generalized to other clinical environments or customized imaging systems. These excluded aspects represent important areas for future research.

## 5. Conclusions

For these publicly accessible LLMs, despite their increasing use in medical applications, the ability to independently and accurately interpret a single CXR still faces some limitations and may require further development. As a tool to assist in clinical image interpretation, addressing issues like side confusion and providing sufficient differential diagnoses during training are crucial. Future studies may explore whether fine-tuning strategies could further improve diagnostic performance, particularly in clinically challenging categories.

## Figures and Tables

**Table 1:** Per-category distribution of cases and interpretation accuracy.

		*n*	Gemini 1.0		Gemini 1.5 Pro		GPT-4 Turbo		GPT-4o	
			Fully	Partial	Fully	Partial	Fully	Partial	Fully	Partial
Acute pulmonary edema		20	7 (35.0%)	7 (35.0%)	6 (30.0%)	11 (55.0%)	0	3 (15.0%)	6 (30.0%)	3 (15.0%)
Cardiomegaly		20	1 (5.0%)	4 (20.0%)	3 (15.0%)	4 (20.0%)	1 (5.0%)	1 (5.0%)	0	0
Lobar pneumonia		15	1 (6.7%)	9 (60.0%)	3 (20.0%)	10 (66.7%)	0	2 (13.3%)	1 (6.7%)	7 (46.7%)
Pacemaker		15	10 (66.7%)	4 (26.7%)	8 (53.3%)	7 (46.7%)	13 (86.7%)	2 (13.3%)	6 (40.0%)	9 (60.0%)
Port-A-Cath		5	0	4 (80.0%)	0	5 (100.0%)	0	5 (100.0%)	2 (40.0%)	3 (60.0%)
PICC		10	0	0	0	0	0	0	0	0
Malignancy (single lesion)		22	0	4 (18.2%)	0	2 (9.1%)	0	1 (4.5%)	1 (4.5%)	1 (4.5%)
Malignancy (multiple lesions)		30	4 (13.3%)	18 (60.0%)	0	20 (66.7%)	1 (3.3%)	8 (26.7%)	5 (16.7%)	10 (33.3%)
Malignancy (central)		15	0	0	0	2 (13.3%)	0	0	0	0
Hiatal hernia		10	0	0	0	0	0	0	0	0
Normal findings		30	28 (93.3%)	0	29 (96.7%)	0	24 (80.0%)	0	30 (100.0%)	0
Diaphragm elevation		5	0	1 (20.0%)	0	1 (20.0%)	0	0	1 (20.0%)	0
Pleural effusion		50	3 (6.0%)	12 (24.0%)	6 (12.0%)	9 (18.0%)	0	3 (6.0%)	4 (8.0%)	11 (22.0%)
	Minimal	15	1 (6.7%)	1 (6.7%)	1 (6.7%)	0	0	0	0	0
	Small	10	0	3 (30.0%)	2 (20.0%)	2 (20.0%)	0	0	0	1 (10.0%)
	Moderate	15	1 (6.7%)	5 (33.3%)	0	6 (40.0%)	0	1 (6.7%)	1 (6.7%)	4 (26.7%)
	Massive	10	1 (10%)	3 (30%)	3 (30%)	1 (10%)	0	2 (20%)	3 (30%)	6 (60%)

Fully, fully correct interpretation; Partial, partially correct interpretation; *n*, total number of cases in each category. Values are presented as counts, with proportions shown in parentheses.

## Data Availability

This study utilized the National Institutes of Health Chest X-ray Dataset (NIH CXR dataset), available for public use under the Creative Commons License ‘CC0: Public Domain.’ This open-access dataset allows scientists unrestricted use for research purposes. In accordance with the requirements, The NIH Clinical Center provided the dataset accessible at: https://nihcc.app.box.com/v/ChestXray-NIHCC (accessed on 21 December 2025), with the reference [32].

## Supplementary Materials

The following supporting information can be downloaded at: https://www.mdpi.com/article/10.3390/diagnostics16030376/s1, Section S1. CLAIM 2024 update; Section S2. Test dataset; Section S3. Overall Primary Diagnosis Performance per Model; Section S4. Statistical Analysis per Group.

## Abbreviations

The following abbreviations are used in this manuscript:

CXRs	Chest X-rays
LLMs	Large language models with vision capabilities
GPT	Generative pretrained transformer
NIHCXRs	National Institutes of Health Chest X-ray Dataset
PICC	Peripherally inserted central catheter
API	Application programming interface
PA/AP	Posterior–anterior or anterior–posterior view
CVC	Central venous catheters
