# Supplementary material for: Limitations in Chest X-Ray Interpretation by Vision-Capable Large Language Models, Gemini 1.0, Gemini 1.5 Pro, GPT-4 Turbo, and GPT-4o
Source: Diagnostics (Basel). 2026 Jan 23;16(3):376. doi: 10.3390/diagnostics16030376 (PMC12897257; doi:10.3390/diagnostics16030376)
Supplement: Supplementary file 1 [file diagnostics-16-00376-s001.zip › diagnostics-4086082-supplementary.pdf]

Supplemental Material

S1. CLAIM 2024 update

The table for CLAIM: 2024 update was downloaded from the website https://pubs.rsna.org/page/ai/claim,
(docx: https://pubs.rsna.org/pb-assets/AI/CLAIM/CLAIMChecklist-6142024-1718376172847.docx), and details on how this manuscript met the requirements were added to the corresponding cells under each item for explanation.

Checklist for Artificial Intelligence in Medical Imaging (CLAIM): 2024 Update

Section / Topic	No	Item	Page / Line	No	NA
TITLE / ABSTRACT					
	**1**	Identification as a study of AI methodology, specifying the category of technology used (e.g., deep learning)	**V**		
ABSTRACT					
	**2**	Summary of study design, methods, results, and conclusions	**V**		
INTRODUCTION					
	**3**	Scientific and/or clinical background, including the intended use and role of the AI approach	**V**		
	**4**	Study aims, objectives, and hypotheses	**V**		
METHODS					
Study Design	**5**	Prospective or retrospective study	**V**		
	A prospective study for LLM in detecting lesion without hints				
	**6**	Study goal	**V**		

		To investigate the interpretation of chest X-rays (CXR) by vision-capable large language models (vLLMs), we analyzed their text outputs to identify current limitations and provide insights for future improvements in their applications.			
Data	**7**	Data sources	**V**		
		NIHCXR dataset, publicly available under the Creative Commons License "CC0: Public Domain."			
	**8**	Inclusion and exclusion criteria	**V**		
		Two pulmonologists independently selected images from the NIH-CXR dataset that clearly corresponded to one of 13 predefined primary diagnoses. Only images with unequivocal findings were included. If either pulmonologist expressed uncertainty regarding a case, that image was excluded from use.			
	**9**	Data pre-processing	**V**		
		No need for data pre-processing			
	**10**	Selection of data subsets	**V**		
		Two pulmonologists selected images from the NIH-CXR dataset that corresponded to 13 predefined primary diagnoses for use in this study.			
	**11**	De-identification methods	**V**		
		The original image files had been fully de-identified. In addition, each file was randomly assigned a new filename ranging from 0 to 246 for anonymized renaming prior to use.			
	**12**	How missing data were handled			**V**
		No missing data issue			
	**13**	Image acquisition protocol	**V**		
		Chest X-ray images were obtained from the publicly accessible NIH Chest X-ray dataset.			
Reference Standard	**14**	Definition of method(s) used to obtain reference standard	**V**		
		These conditions—including acute pulmonary edema, cardiomegaly, hiatal			

	hernia, lobar pneumonia, pacemaker device, port-a-cath device, peripherally inserted central catheter, pleural effusion with minimal, small, moderate, and massive amounts, malignancy with centrally distributed, single, or multiple lesions, diaphragm elevation, and no significant findings—are all well-defined entities in clinical chest X-ray interpretation.				
	**15**	Rationale for choosing the reference standard	**V**		
	The images were selected by two pulmonologists, and only those chest X-rays (CXRs) on which both reviewers reached full consensus without ambiguity were included for analysis.				
	**16**	Source of reference standard annotations	**V**		
	Images were selected by two pulmonologists based on clearly distinguishable and unequivocal radiographic features, while minimizing the inclusion of unnecessary or confounding CXR findings.				
	**17**	Annotation of test set	**V**		
	The test set was randomized, and the correspondence between the test filenames, original filenames, and primary diagnoses was used for labeling.				
	**18**	Measures of inter- and intra-rater variability of features described by the annotators			**V**
	Only images with unequivocal findings were included. If either pulmonologist expressed uncertainty regarding a case, that image was excluded from use.				
Data Partitions	**19**	How data were assigned to partitions			**V**
	The entire test dataset was used in the analysis; therefore, no data partitioning was performed.				
	**20**	Level at which partitions are disjoint			**V**
	No issue of disjoint partitions was present in this study.				
Testing Data	**21**	Intended sample size	**V**		
	Total 247 images with 13 categories of primary diagnoses				

S2. Test dataset

Examples Images of 13 Categories (except normal findings)

Acute lung edema	Lobar pneumonia	Cadiomegaly
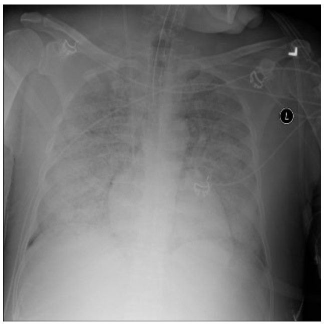	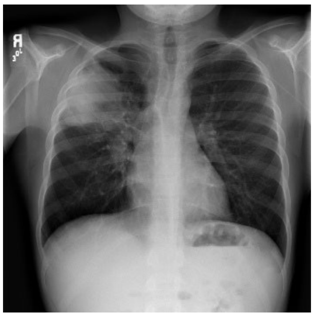	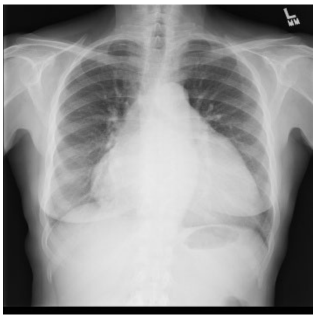
00001787_005	00001583_000(rt)	00000211_006

M*-Single	M*-multiple
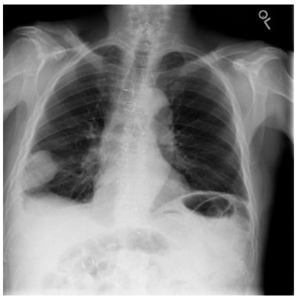	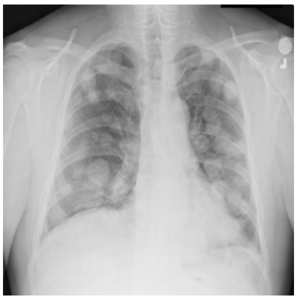
00000012_000(rt)	00004222_000

M*-central	Hiatal hernia
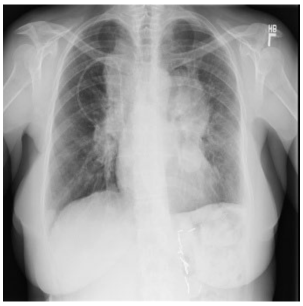	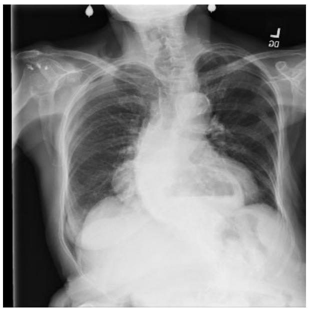
00002661_001	00000284_005

Pacemaker	Port-a-cath	PICC
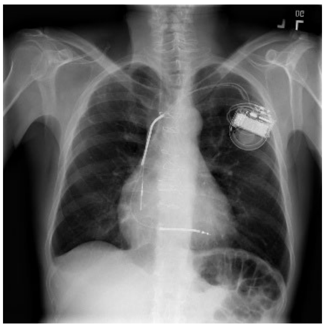	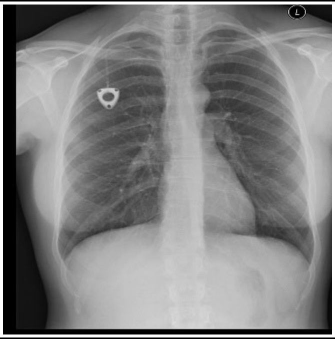	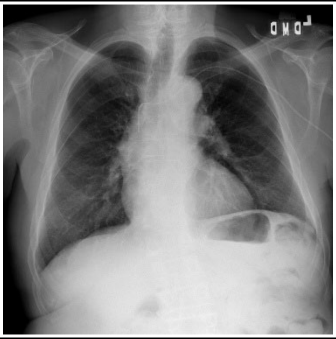
00000013_044(lt)	00000651_000(rt)	00000039_003(lt)

*M: malignancy

Examples of pleural effusion and diaphragm elevation

Minimal	Small	Moderate	Massive	Diaphragm Elevation
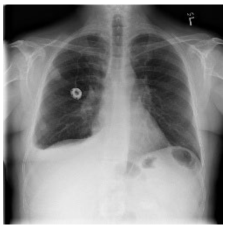	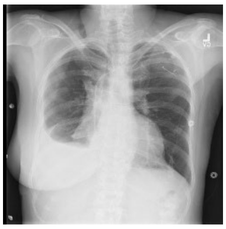	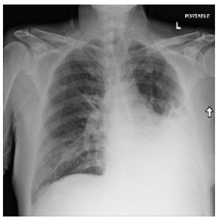	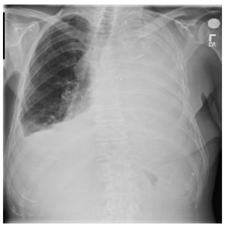	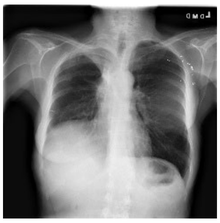
00000148_001	00002464_003(rt)	00000467_001(lt)	00000025_000(lt)	00000090_010

The files in the test dataset were randomly renamed from selected images of the NIH Chest X-ray (NIH-CXR) collection. For easier reference during interpretation, the laterality of the lesions (right or left) was indicated in parentheses for cases with side-specific findings on the CXR. The correspondence between each new test filename, its original filename, and the primary diagnosis is summarized in the following table.

Num	Test filename	Original filename	Primary diagnosis
1	00000000.png	00000121_008(rt).png	PICC
2	00000001.png	00004381_006.png	cardiomegaly
3	00000002.png	00000167_001(rt).png	porta
4	00000003.png	00003751_003(rt).png	single lesion
5	00000004.png	00000111_000.png	hiatal hernia
6	00000005.png	00000155_000.png	cardiomegaly
7	00000006.png	00000457_002.png	minimal pe
8	00000007.png	00000046_000(lt).png	pacemaker
9	00000008.png	00002413_005(rt).png	massive pe
10	00000009.png	00000942_000.png	hiatal hernia
11	00000010.png	00001583_000(rt).png	lobar pneumonia
12	00000011.png	00005221_002.png	multiple lesion
13	00000012.png	00002413_004(rt).png	massive pe
14	00000013.png	00000441_000.png	diaphragm elevation
15	00000014.png	00000662_007.png	no finding
16	00000015.png	00005793_001.png	central lesion
17	00000016.png	00002224_005.png	ac pul edema
18	00000017.png	00001158_001(rt).png	single lesion
19	00000018.png	00001900_018.png	central lesion

20	00000019.png	00006414_001.png	multiple lesion
21	00000020.png	00003254_001(rt).png	moderate pe
22	00000021.png	00000108_000(lt).png	pacemaker
23	00000022.png	00005626_002(rt).png	moderate pe
24	00000023.png	00004222_000.png	multiple lesion
25	00000024.png	00001483_018(rt).png	lobar pneumonia
26	00000025.png	00000025_000(lt).png	massive pe
27	00000026.png	00002785_006(rt).png	lobar pneumonia
28	00000027.png	00000979_005(rt).png	lobar pneumonia
29	00000028.png	00004850_008.png	minimal pe
30	00000029.png	00000087_001(lt).png	PICC
31	00000030.png	00004526_024.png	cardiomegaly
32	00000031.png	00004583_000(rt).png	moderate pe
33	00000032.png	00003973_001.png	cardiomegaly
34	00000033.png	00001443_000.png	no finding
35	00000034.png	00002390_003(rt).png	moderate pe
36	00000035.png	00001494_000(lt).png	pacemaker
37	00000036.png	00003426_038(lt).png	small pe
38	00000037.png	00004317_000.png	ac pul edema
39	00000038.png	00005337_003(bil).png	small pe
40	00000039.png	00003534_037.png	ac pul edema
41	00000040.png	00001075_024.png	central lesion
42	00000041.png	00001456_001.png	no finding
43	00000042.png	00001508_000.png	no finding
44	00000043.png	00006480_002.png	minimal pe
45	00000044.png	00005632_002.png	multiple lesion
46	00000045.png	00004436_006.png	multiple lesion
47	00000046.png	00004344_037.png	ac pul edema
48	00000047.png	00005113_004.png	minimal pe
49	00000048.png	00003186_005.png	ac pul edema
50	00000049.png	00000363_002.png	minimal pe
51	00000050.png	00000442_001.png	cardiomegaly
52	00000051.png	00000469_001.png	no finding
53	00000052.png	00002510_003.png	multiple lesion
54	00000053.png	00003940_002.png	ac pul edema
55	00000054.png	00004281_003.png	central lesion
56	00000055.png	00003719_010(rt).png	small pe

57	00000056.png	00000512_002.png	cardiomegaly
58	00000057.png	00004740_000(lt).png	pacemaker
59	00000058.png	00002613_002.png	multiple lesion
60	00000059.png	00000450_008(rt).png	PICC
61	00000060.png	00000280_001.png	no finding
62	00000061.png	00005854_003.png	minimal pe
63	00000062.png	00000315_000(rt).png	lobar pneumonia
64	00000063.png	00000410_001(lt).png	pacemaker
65	00000064.png	00000263_000(rt).png	pacemaker
66	00000065.png	00002599_001(rt).png	lobar pneumonia
67	00000066.png	00003186_002.png	ac pul edema
68	00000067.png	00000561_003.png	no finding
69	00000068.png	00000284_005.png	hiatal hernia
70	00000069.png	00000129_001.png	no finding
71	00000070.png	00004592_000.png	multiple lesion
72	00000071.png	00004117_000(lt).png	single lesion
73	00000072.png	00005895_007(rt).png	moderate pe
74	00000073.png	00001101_004(rt).png	small pe
75	00000074.png	00003766_001.png	minimal pe
76	00000075.png	00006308_003.png	multiple lesion
77	00000076.png	00000728_000.png	cardiomegaly
78	00000077.png	00000148_001.png	minimal pe
79	00000078.png	00003938_000.png	hiatal hernia
80	00000079.png	00002437_003.png	ac pul edema
81	00000080.png	00005410_000.png	central lesion
82	00000081.png	00000782_002(lt).png	pacemaker
83	00000082.png	00006010_000(lt).png	single lesion
84	00000083.png	00005505_000.png	multiple lesion
85	00000084.png	00001480_000(lt).png	porta
86	00000085.png	00004104_000.png	central lesion
87	00000086.png	00003426_021(lt).png	massive pe
88	00000087.png	00001301_012.png	ac pul edema
89	00000088.png	00006338_001(rt).png	single lesion
90	00000089.png	00001418_000.png	no finding
91	00000090.png	00002437_027.png	ac pul edema
92	00000091.png	00003593_004(lt).png	small pe
93	00000092.png	00000415_002(rt).png	PICC

94	00000093.png	00004656_000(rt).png	single lesion
95	00000094.png	00002785_005(rt).png	lobar pneumonia
96	00000095.png	00005970_007(lt).png	single lesion
97	00000096.png	00002051_000(rt).png	lobar pneumonia
98	00000097.png	00001431_000.png	no finding
99	00000098.png	00005298_012.png	multiple lesion
100	00000099.png	00000090_010.png	diaphragm elevation
101	00000100.png	00006518_000.png	central lesion
102	00000101.png	00000587_000(lt).png	pacemaker
103	00000102.png	00000259_000.png	no finding
104	00000103.png	00005395_000(lt).png	single lesion
105	00000104.png	00000428_000(lt).png	pacemaker
106	00000105.png	00001637_007.png	central lesion
107	00000106.png	00000042_002(rt).png	PICC
108	00000107.png	00004750_006(rt).png	single lesion
109	00000108.png	00000467_001(lt).png	moderate pe
110	00000109.png	00003163_001(lt).png	pacemaker
111	00000110.png	00002464_003(rt).png	small pe
112	00000111.png	00000234_000(rt).png	porta
113	00000112.png	00000661_000.png	cardiomegaly
114	00000113.png	00003803_010.png	ac pul edema
115	00000114.png	00003083_002(rt).png	small pe
116	00000115.png	00002229_002.png	minimal pe
117	00000116.png	00005794_002.png	central lesion
118	00000117.png	00002533_003.png	ac pul edema
119	00000118.png	00003957_002(lt).png	moderate pe
120	00000119.png	00000410_000(lt).png	pacemaker
121	00000120.png	00002361_010.png	multiple lesion
122	00000121.png	00001450_000.png	no finding
123	00000122.png	00000143_003.png	no finding
124	00000123.png	00002227_002.png	minimal pe
125	00000124.png	00003547_000.png	multiple lesion
126	00000125.png	00006330_003(rt).png	single lesion
127	00000126.png	00002688_005.png	ac pul edema
128	00000127.png	00003338_000.png	cardiomegaly
129	00000128.png	00006072_000.png	multiple lesion
130	00000129.png	00000162_000(lt).png	pacemaker

131	00000130.png	00000877_035.png	diaphragm elevation
132	00000131.png	00003875_006.png	ac pul edema
133	00000132.png	00004911_000(lt).png	single lesion
134	00000133.png	00000229_000.png	no finding
135	00000134.png	00001205_002.png	hiatal hernia
136	00000135.png	00004532_000(lt).png	single lesion
137	00000136.png	00000296_003.png	no finding
138	00000137.png	00005628_000.png	multiple lesion
139	00000138.png	00001583_001(rt).png	lobar pneumonia
140	00000139.png	00000385_001.png	hiatal hernia
141	00000140.png	00000001_002.png	cardiomegaly
142	00000141.png	00006192_012(rt).png	small pe
143	00000142.png	00005912_004.png	minimal pe
144	00000143.png	00006558_000.png	multiple lesion
145	00000144.png	00000459_041.png	ac pul edema
146	00000145.png	00001582_023.png	cardiomegaly
147	00000146.png	00006198_000.png	multiple lesion
148	00000147.png	00001437_053(rt).png	massive pe
149	00000148.png	00003005_005(lt).png	massive pe
150	00000149.png	00001518_000.png	no finding
151	00000150.png	00000012_000(rt).png	single lesion
152	00000151.png	00000003_006.png	hiatal hernia
153	00000152.png	00001517_010(lt).png	PICC
154	00000153.png	00002104_000(rt).png	lobar pneumonia
155	00000154.png	00001301_023(lt).png	massive pe
156	00000155.png	00005762_009(rt).png	small pe
157	00000156.png	00005578_002.png	central lesion
158	00000157.png	00000409_000.png	minimal pe
159	00000158.png	00000013_044(lt).png	pacemaker
160	00000159.png	00004519_015.png	multiple lesion
161	00000160.png	00003725_004(rt).png	moderate pe
162	00000161.png	00005722_024.png	ac pul edema
163	00000162.png	00004094_001.png	central lesion
164	00000163.png	00000096_001.png	cardiomegaly
165	00000164.png	00005977_015.png	multiple lesion
166	00000165.png	00001466_000.png	no finding
167	00000166.png	00000627_001.png	diaphragm elevation

168	00000167.png	00005066_046.png	cardiomegaly
169	00000168.png	00003744_000.png	cardiomegaly
170	00000169.png	00000057_000.png	no finding
171	00000170.png	00003400_000.png	multiple lesion
172	00000171.png	00000395_000(lt).png	pacemaker
173	00000172.png	00005318_000(lt).png	single lesion
174	00000173.png	00001214_000.png	central lesion
175	00000174.png	00001451_000.png	no finding
176	00000175.png	00000250_012(rt).png	PICC
177	00000176.png	00003725_005(rt).png	moderate pe
178	00000177.png	00001787_005.png	ac pul edema
179	00000178.png	00006302_001.png	multiple lesion
180	00000179.png	00004514_002(rt).png	massive pe
181	00000180.png	00000651_000(rt).png	porta
182	00000181.png	00000211_006.png	cardiomegaly
183	00000182.png	00005760_000(rt).png	single lesion
184	00000183.png	00000347_000(lt).png	pacemaker
185	00000184.png	00001458_003(rt).png	PICC
186	00000185.png	00004291_001.png	cardiomegaly
187	00000186.png	00004132_009.png	multiple lesion
188	00000187.png	00000445_000.png	no finding
189	00000188.png	00006378_005.png	hiatal hernia
190	00000189.png	00001480_001(lt).png	porta
191	00000190.png	00001517_000(rt).png	PICC
192	00000191.png	00003284_000(rt).png	single lesion
193	00000192.png	00005532_024.png	cardiomegaly
194	00000193.png	00000531_005.png	no finding
195	00000194.png	00005679_005(rt).png	single lesion
196	00000195.png	00003064_039(lt).png	moderate pe
197	00000196.png	00000266_000.png	no finding
198	00000197.png	00004344_014.png	cardiomegaly
199	00000198.png	00000480_000.png	no finding
200	00000199.png	00004889_000(lt).png	single lesion
201	00000200.png	00005712_026(rt).png	lobar pneumonia
202	00000201.png	00001509_000.png	no finding
203	00000202.png	00005030_000.png	multiple lesion
204	00000203.png	00000037_000.png	no finding

205	00000204.png	00002412_006(rt).png	lobar pneumonia
206	00000205.png	00003737_001.png	multiple lesion
207	00000206.png	00000963_010.png	minimal pe
208	00000207.png	00000116_006(lt).png	massive pe
209	00000208.png	00004299_011.png	ac pul edema
210	00000209.png	00002490_000.png	minimal pe
211	00000210.png	00006182_000.png	multiple lesion
212	00000211.png	00000691_000.png	no finding
213	00000212.png	00000348_001.png	no finding
214	00000213.png	00000312_000.png	no finding
215	00000214.png	00000506_001(lt).png	massive pe
216	00000215.png	00001470_003.png	diaphragm elevation
217	00000216.png	00000252_001.png	no finding
218	00000217.png	00000554_000.png	no finding
219	00000218.png	00002275_016(rt).png	lobar pneumonia
220	00000219.png	00003528_017.png	ac pul edema
221	00000220.png	00004872_000.png	multiple lesion
222	00000221.png	00001719_011(rt).png	lobar pneumonia
223	00000222.png	00002661_001.png	central lesion
224	00000223.png	00003837_001.png	central lesion
225	00000224.png	00002552_002.png	ac pul edema
226	00000225.png	00004738_004.png	multiple lesion
227	00000226.png	00000040_003(lt).png	single lesion
228	00000227.png	00001410_001.png	multiple lesion
229	00000228.png	00002089_000.png	hiatal hernia
230	00000229.png	00001808_000(lt).png	single lesion
231	00000230.png	00000742_001(rt).png	moderate pe
232	00000231.png	00001558_034(lt).png	moderate pe
233	00000232.png	00006273_001.png	multiple lesion
234	00000233.png	00002843_010.png	minimal pe
235	00000234.png	00003826_001(lt).png	moderate pe
236	00000235.png	00000103_001.png	central lesion
237	00000236.png	00005895_013(rt).png	moderate pe
238	00000237.png	00002763_012.png	cardiomegaly
239	00000238.png	00003519_005.png	multiple lesion
240	00000239.png	00003029_008.png	hiatal hernia
241	00000240.png	00004843_000.png	cardiomegaly

242	00000241.png	00004602_001(lt).png	single lesion
243	00000242.png	00000039_003(lt).png	PICC
244	00000243.png	00006425_007(lt).png	lobar pneumonia
245	00000244.png	00005695_000(rt).png	moderate pe
246	00000245.png	00003470_000(rt).png	small pe
247	00000246.png	00002702_000(lt).png	single lesion

S3. Overall Primary Diagnosis Performance per Model

Model	Fully correct n (%)	95% CI	Fully + Partially correct n (%)	95% CI
Gemini 1.0	54 (21.9%)	17.2–27.4%	117 (47.4%)	41.2–53.6%
Gemini 1.5 Pro	55 (22.3%)	17.5–27.9%	126 (51.0%)	44.8–57.2%
GPT-4 Turbo	39 (15.8%)	11.8–20.9%	64 (25.9%)	20.8–31.7%
GPT-4o	56 (22.7%)	17.9–28.3%	100 (40.5%)	34.6–46.7%
	$\chi^2 = 4.79$, $p = 0.188$		$\chi^2 = 37.58$, $p < 0.0001$	

S4. Statistical Analysis per Group

Imaging Feature Group	Subcategory	Fully Correct (%)	χ^2	p value	Detection Rate (%)	χ^2	p value
Large-sized lesions	Acute pulmonary edema	23.8	12.4	0.0020	53.8	28.52	<0.0001
	Lobar pneumonia	8.3			55.0		
	Cardiomegaly	6.2			17.5		
Lesion number	Malignancy, multiple lesions	8.3	3.91	0.0480	55.0	42.22	<0.0001
	Malignancy, single lesion	1.1			10.2		
Mediastinal involvement	Central malignancy	0.0	-	-	0.0	-	-
	Hiatal hernia	0.0			0.0		
Devices	Pacemaker	61.7	47.14	<0.0001	98.3	111.50	<0.0001
	Port-a-cath	10.0			95.0		
	PICC	0.0			0.0		

	Massive	17.5	10.36	0.0348	47.5	26.97	<0.0001
	Moderate	3.3			30.0		
Pleural findings	Small	5.0			20.0		
	Minimal	3.3			5.0		
	Diaphragm elevation	5.0			15.0		

Large-sized lesions: acute pulmonary edema, lobar pneumonia, cardiomegaly

Acute pulmonary edema affects both lung fields, while lobar pneumonia involves a localized lesion within a single lung field. Cardiomegaly, on the other hand, is located within the mediastinum and partially obscures the medial areas of both lower lung fields. These categories were used to evaluate the model's ability to detect lesions of different sizes.

The combined results of four vision-capable large language models revealed significant differences in diagnostic performance across the three representative conditions. When only fully correct responses were considered, the chi-square test indicated a statistically significant difference among the three categories (χ^2 = 12.40, p = 0.0020). The highest fully correct rate was observed for Acute pulmonary edema (23.8%), followed by Lobar pneumonia (8.3%), and the lowest for Cardiomegaly (6.2%). When both fully and partially correct responses were considered as successful detections, the difference remained significant (χ^2 = 28.52, p = 0.0000). The detection rates followed a similar pattern, ranking as Lobar pneumonia (55.0%), Acute pulmonary edema (53.8%), and Cardiomegaly (17.5%).

These findings indicate that the vision-capable LLMs exhibited higher sensitivity to large or diffuse pulmonary lesions than to mediastinal enlargement. Overall performance ranking: Fully correct → Acute pulmonary edema > Lobar pneumonia > Cardiomegaly; Detection (Fully + Partial) → Lobar pneumonia > Acute pulmonary edema > Cardiomegaly.

Lesion number: malignancy with single lesion vs. multiple lesions

This comparison was used to assess the model's sensitivity to the number of lesions present within the image. The combined results of four vision-capable large language models revealed

significant differences in diagnostic performance across the three representative conditions. When only fully correct responses were considered, the chi-square test indicated a statistically significant difference among the three categories (χ^2 = 3.911544, p = 0.047956). The fully correct rates were Multiple (8.3%), and Single (1.1%). When both fully and partially correct responses were considered as successful detections, the difference remained significant (χ^2 = 42.220382, p = 0.000000). The detection rates were Multiple (55.0%), and Single (10.2%).

These findings indicate that the vision-capable LLMs exhibited higher sensitivity to large or diffuse pulmonary lesions than to mediastinal enlargement. Overall performance ranking: Fully correct → Multiple > Single; Detection (Fully + Partial) → Multiple > Single.

Mediastinum: Malignancy with central distribution and hiatal hernia

These categories were selected to evaluate the model's detection capability for lesions located in or adjacent to the mediastinum. Across all four vLLMs, both "malignancy with central distribution" and "hiatal hernia" demonstrated complete failure of recognition. None of the models produced any fully correct responses, and all models yielded zero detections for hiatal hernia.

For malignancy with central distribution, only two partially correct responses were generated by Gemini 1.5 Pro, without any fully correct answers. Because all observed counts were zero or near-zero, no valid chi-square test could be performed. These findings indicate that all models exhibited no detectable capability to recognize lesions located within or adjacent to the mediastinum in these categories.

Devices: pacemaker, port-a-cath, and PICC

These devices have distinct appearances and strong radiographic contrasts. We used pacemaker, port-a-cath, and peripherally inserted central catheter (PICC) for evaluation. Among them, the pacemaker is larger than the port-a-cath, and both exhibit line-like structures on chest X-rays, whereas the PICC primarily represents a linear structure alone.

The combined results of four vision-capable large language models revealed significant differences in diagnostic performance across the three representative conditions. When only fully correct responses were considered, the chi-square test indicated a statistically significant difference among the three categories ($\chi^2 = 47.141500$, $p = 0.000000$). The highest fully correct rate was observed for pacemaker (61.7%), followed by port-a-cath (10.0%), and the lowest for PICC (0.0%). When both fully and partially correct responses were considered as successful detections, the difference remained significant ($\chi^2 = 111.501832$, $p = 0.000000$). The detection rates followed a similar pattern, ranking as pacemaker (98.3%), port-a-cath (95.0%), and PICC (0.0%).

These findings indicate that the vision-capable LLMs exhibited higher sensitivity to large or diffuse pulmonary lesions than to mediastinal enlargement. Overall performance ranking: Fully Correct → Pacemaker > Port-a-cath > PICC; Detection (Fully + Partial) → Pacemaker > Port-a-cath > PICC.

Pleural effusion: minimal, small, moderate, and massive and diaphragm elevation

These categories were included to assess the model's ability to detect pleural effusion. Diaphragm elevation shares radiographic features similar to small or moderate pleural effusion, although they are pathophysiologically distinct conditions.

The combined results of four vision-capable large language models revealed significant differences in diagnostic performance across the three representative conditions. When only fully correct responses were considered, the chi-square test indicated a statistically significant difference among the three categories ($\chi^2 = 10.361766$, $p = 0.034755$). The fully correct rates were Massive (17.5%), Small (5.0%), Diaphragm Elevation (5.0%), Minimal (3.3%), and Moderate (3.3%). When both fully and partially correct responses were considered as successful detections, the difference remained significant ($\chi^2 = 26.967166$, $p = 0.000020$). The detection rates were Massive (47.5%), Moderate (30.0%), Small (20.0%), Diaphragm Elevation (15.0%), and Minimal (5.0%).

These findings indicate that the vision-capable LLMs exhibited higher sensitivity to large or diffuse pulmonary lesions than to mediastinal enlargement. Overall performance ranking: Fully correct → Massive > Small > Diaphragm Elevation > Minimal > Moderate; Detection (Fully + Partial) → Massive > Moderate > Small > Diaphragm Elevation > Minimal.
